# Acute myocardial infarction in an 18 year old South Indian girl with familial hypercholesterolemia: a case report

**DOI:** 10.1186/1757-1626-1-71

**Published:** 2008-08-07

**Authors:** Anita A Kumar, Ghanshyam Palamaner Subash Shantha, Yadav Srinivasan, N Senthil, K Rajkumar, Neeta Paunikar, MK Sudhakar

**Affiliations:** 1 Department of General Medicine, Sri Ramachandra University, Chennai, India

## Abstract

Familial hypercholesterolemia is a single gene disorder with an autosomal dominant pattern of inheritance. Here we report an 18 year old South Indian girl who presented with myocardial infarction. She had xanthomas and an elevated serum low density lipoprotein cholesterol (LDL-C). Her mother and maternal uncle had died at a young age due to myocardial infarction. Her only sibling, 15 year old younger sister also had xanthomas and an elevated LDL-C. This report is to emphasise the need to clinically recognize xanthomas and its association with elevated LDL-C, premature atherosclerosis and familial inheritance. Early diagnosis and early initiation of treatment will save the affected individual and the other family members.

## Background

Muller et el in the year 1938 reported a familial clustering of xanthoma, high cholesterol and myocardial infarction [1]. In 1960, Khachadurian et al demonstrated that this clinical association termed familial hypercholesterolemia (FH) is a single gene disorder with an autosomal dominant pattern of inheritance [2]. Further studies identified the association between improper metabolism of LDL and FH and that mutation in the LDL receptor gene (LDLR) located on chromosome 19 was responsible for this disorder [3-5]. FH is of 2 types, a homozygous type where both LDL receptor alleles are defective due to mutation and has a prevalence of 1 in 1 million persons world wide [6]. In the heterozygous type only one LDL receptor allele is a mutant and is much more common with a prevalence of 1 in 500 persons worldwide [6]. Here we report one such family from South India with features of premature atherosclerosis, high serum levels of LDL cholesterol and multiple xanthomas.

## Case presentation

**Figure 1:**
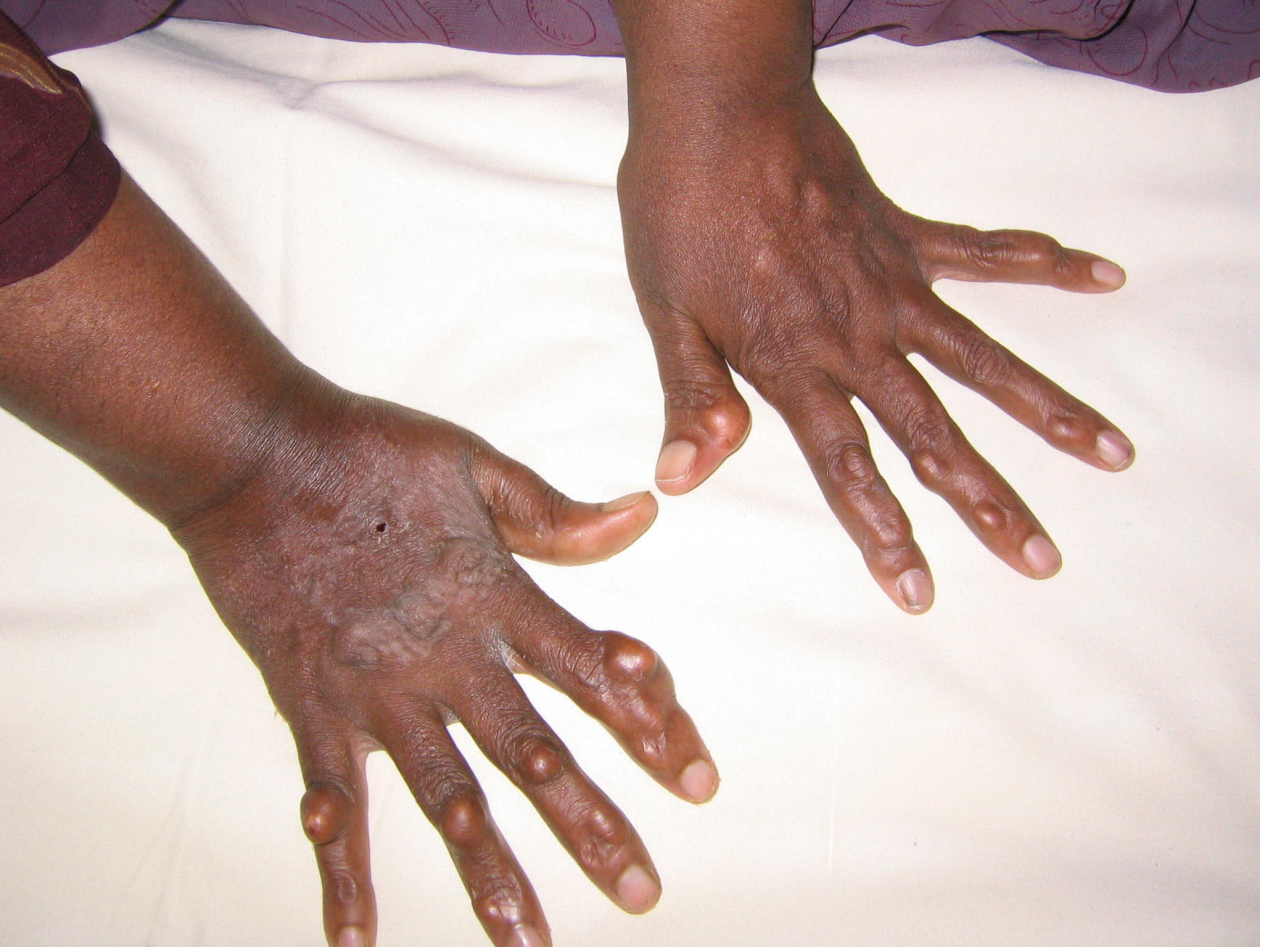
**Xanthomas**. Photograph of patient's hands showing multiple xanthomas.

**Figure 2:**
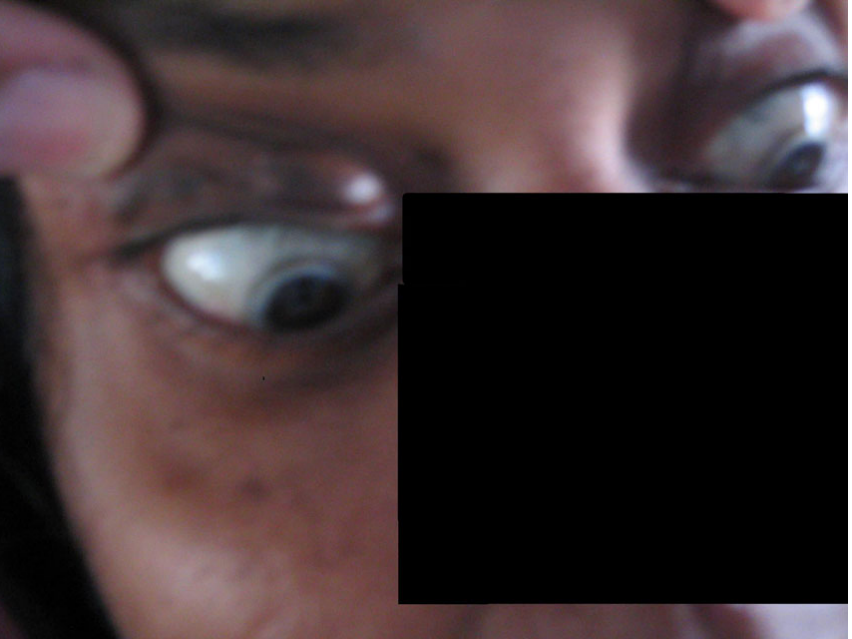
**Corneal arcus**. Photograph of patient's eyes showing corneal arcus, white rim coating the limbus in both eyes.

**Figure 3:**
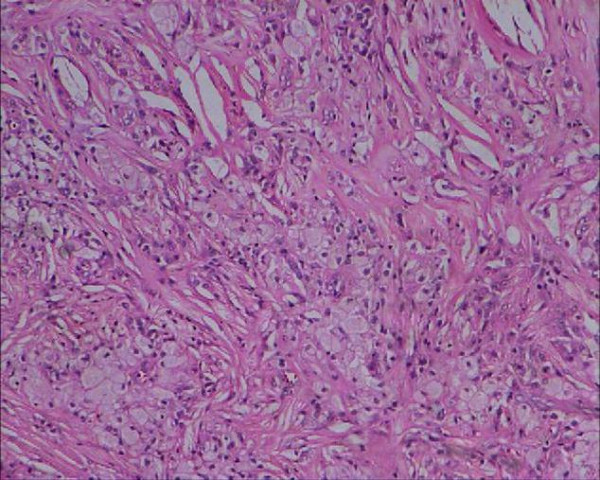
**Histology of Xanthoma**. Histology picture of xanthoma showing lipid laden foam cells with large areas of cholesterol clefts, 10 × magnification, eosin and hematoxilin stain.

**Figure 4:**
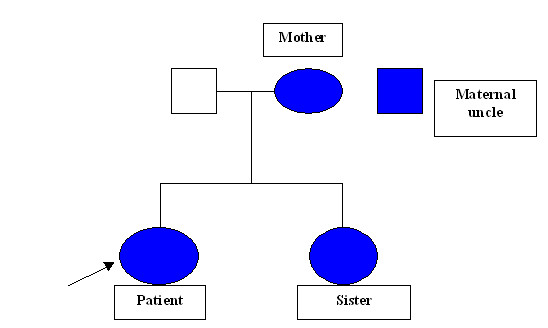
Pedigree chart.

An 18 year old South Indian girl presented to the emergency department of a tertiary care hospital with complaints of anginal chest pain and dyspnea for 4 hours duration. She was a tailor by occupation. She had no past history of diabetes mellitus, hypothyroidism, coronary artery disease, hepatic disease or renal disease. She was unmarried and had regular menstrual cycles. She weighed 54 kgs and was 157 cms tall. Her body mass index was 22 kg/m2. General physical examination revealed multiple soft subcutaneous swellings over her hand, knuckles, achilles tendon, wrist and ankle suggestive of xanthomas (Figure 1). She had corneal arcus (Figure 2). Her systems exam revealed tachycardia, S3 gallop on cardiac auscultation and bibasilar crackles in the lungs. Electrocardiogram was consistent with acute anterior wall myocardial infarction. Echocardiogram showed a hypokinetic anterior wall of the heart, with a left ventricular ejection fraction of 30%, aortic valves were normal with no evidence of valvular stenosis. She was consequently thrombolysed with tissue plasminogen activator (TPA) for her acute anterior wall myocardial infarction and her pulmonary edema was managed. Her labs revealed normal blood counts and renal functions. Her fasting lipid profile (table 1) showed a high total cholesterol and LDL cholesterol with normal triglycerides. Her angiogram showed 60% stenosis of the left anterior descending coronary artery and a 30% stenosis of the left circumflex coronary artery. The biopsy of one of the swellings (the swelling in her wrist) revealed lipid laden foam cells with large areas of cholesterol clefts (figure 3), suggestive of a xanthoma. On further questioning she gave a family history of accelerated atherosclerosis. Her mother and her maternal uncle had died due to myocardial infarction at the age of 23 years and 19 yrs respectively and that her mother also had similar xanthomas. Her only sibling her younger sister (15 yrs old) in addition to having xanthomas, had a lipid profile (Table 1) consistent with a high total cholesterol and LDL cholesterol with normal triglycerides. Her pedigree chart can be seen in Figure 4.

**Table 1:** Fasting lipid profile values of the patient and her sister

	Patient's values		Patient's sister's values	
	mg/dl	mmol/l	mg/dl	mmol/l

Total cholesterol	657 mg/dl	16.8 mmol/l	630 mg/dl	16.15 mmol/l
LDL-C	572 mg/dl	14.6 mmol/l	554 mg/dl	14.2 mmol/l
HDL-C	61 mg/dl	1.6 mmol/l	50 mg/dl	1.3 mmol/l
Triglycerides	122 mg/dl	1.4 mmol/l	130 mg/dl	1.5 mmol/l

She was initially treated with atorvastatin 80 mg once daily with ezetimibe 10 mg daily. Her LDL-C levels measured after 8 weeks was still 538 mg/dl (table 2). Hence in view of persistent LDL-C elevation inspite of maximal drug therapy for 8 weeks, the patient was advised LDL apheresis and was referred to a specialist center. On follow up, after 3 sessions of LDL apheresis (one session every week), her LDL-C levels were 165 mg/dl (table 2). In addition to weekly LDL apheresis she is also treated with atorvastatin 40 mg and ezetimibe 10 mg daily.

**Table 2:** Patient's lipid levels after treatment

	After 8 weeks therapy with maximal lipid lowering drugs	After 3 sessions of LDL apheresis
Total cholesterol	617 mg/dl	230 mg/dl
LDL-C	538 mg/dl	165 mg/dl
HDL-C	52 mg/dl	41 mg/dl
Triglycerides	133 mg/dl	120 mg/dl

## Discussion

Our patient had myocardial infarction at a young age of 18 yrs with a lipid profile comprising a high LDL-C with normal triglycerides. She had xanthomas and corneal arcus. Her family history was classical with 2 generations being affected with premature atherosclerosis and both male and female individuals being affected equally. Hence this suggests an autosomal dominant disorder. Simon Broome's diagnostic criteria for familial hypercholesterolemia says a definite diagnosis of familial hypercholesterolemia can be made if either the total cholesterol concentration is above 7.5 mmol/liter in adults or the low density lipoprotein cholesterol concentration is above 4.9 mmol/liter in adults and if tendinous xanthomas were present in the patient or a first-degree relative [7,8]. Our patient had total cholesterol of 16.8 mmol/l and a LDL-C of 14.6 mmol/l. She, her mother and her sister had xanthomas. Hence this confirms the diagnosis of familial hypercholesterolemia in our patient. She should have had the homozygous type of FH as the heterozygous type presents much later in adult life with coronary artery disease and LDL-C levels are generally less than 400 mg/dl [6].

Treatment options available for homozygous FH are lipid lowering drugs like statins, bile acid sequestrants, apheresis and liver transplantation [6]. Lipid lowering drugs only result in modest reduction in LDL-C levels and apheresis is generally required when evidence of atherosclerosis is present. As liver is the most important tissue for removing circulating LDL, liver transplantation is an effective treatment option in this disorder. Our patient's LDL-C was refractory to drug therapy and hence she was initiated on LDL apheresis. Her response was satisfactory to this modality of treatment.

## Conclusion

Clinical identification of xanthomas and knowledge of its association with coronary artery disease is essential for every physician as early diagnosis and treatment can prevent premature deaths due to MI. Also all the relatives in the family should be screened for dyslipidemia.

## Patient's perspective

I have been having these swellings in my body for the last 7 years. I would have visited many doctors for various common ailments. Had they recognized this disorder in me much earlier I would not have had a heart attack. Atleast my sister will benefit as she has been diagnosed to have this disease and treatment will soon be initiated. After I get married I will also be careful with my children as I understand that they are also prone for this disease.

## Abbreviations

FH: Familial hypercholesterolemia; LDL-C: Low Density Lipoprotein cholesterol; LDLR: Low Density Lipoprotein Receptor gene; TPA: Tissue Plasminogen Activator; MI: Myocardial Infarction.

## Competing interests

The authors declare that they have no competing interests.

## Authors' contributions

AAK, GPSS, YS, RK were involved in the patient care, acquisition of data, analysis and interpretation of data, review of literature, drafting and revising the manuscript. NS, NP, MKS revised the manuscript for important intellectual content. All authors read and approved the final manuscript.

## Consent

Written informed consent was obtained from the patient for publication of this case report and accompanying images. A copy of the written consent is available for review by the Editor-in-Chief of this journal.
